# A screen of apoptosis and senescence regulatory genes for life span effects when over-expressed in Drosophila

**DOI:** 10.18632/aging.100018

**Published:** 2009-01-30

**Authors:** Jie Shen, Christina Curtis, Simon Tavaré, John Tower

**Affiliations:** ^1^ Molecular and Computational Biology Program, Department of Biological Sciences, University of Southern California, Los Angeles, CA 90089, USA; ^2^ Department of Oncology, University of Cambridge, Cancer Research UK Cambridge Research Institute Li Ka Shing Centre, Robinson Way, Cambridge CB2 0RE, UK

**Keywords:** geneswitch, Wnt, wingless, Ras

## Abstract

Conditional expression of transgenes in *Drosophila* was produced using the Geneswitch system, wherein feeding the drug RU486/Mifepristone activates the artificial transcription factor Geneswitch. Geneswitch was expressed using the *Actin5C* promoter and this was found to yield conditional, tissue-general expression of a target transgene (UAS-GFP) in both larvae and adult flies.  Nervous system-specific (Elav-GS) and fat body-specific Geneswitch drivers were also characterized using UAS-GFP.  Fourteen genes implicated in growth, apoptosis and senescence regulatory pathways were over-expressed in adult flies or during larval development, and assayed for effects on adult fly life span. Over-expression of a dominant *p53* allele (*p53-259H*) in adult flies using the ubiquitous driver produced increased life span in females but not males, consistent with previous studies.  Both *wingless* and *Ras activated form *transgenes were lethal when expressed in larvae, and reduced life span when expressed in adults, consistent with results from other model systems indicating that the *wingless* and *Ras* pathways can promote senescence.  Over-expression of the caspase inhibitor *baculovirus p35* during larval development reduced the mean life span of male and female adults, and also produced a subset of females with increased life span.  These experiments suggest that* baculovirus p35* and the *wingless* and *Ras* pathways can have sex-specific and developmental stage-specific effects on adult *Drosophila* life span, and these reagents should be useful for the further analysis of the role of these conserved pathways in aging.

## Introduction

A number of stresses can cause cells to enter a non-dividing state called cellular senescence [1].  These stresses include repeated cell division, expression of activated oncogenes, oxidative stress, and irradiation. The cellular senescence pathway functions as an anti-tumor mechanism in mammals, and is regulated by the tumor-suppressor proteins p53 and Rb.  Senescence of cells during aging may contribute to mammalian aging phenotypes by limiting the ability of stem cell  populations to replenish tissues.  Several *Drosophila* tissues are maintained by dividing stem cell populations, including the gonads [2], the gut [3,4] and the malpighian tubule (equivalent to mammalian kidney) [5], however it is currently unknown whether alterations in these stem cell populations during aging has an effect on *Drosophila* life span.

Apoptosis (programmed cell death) is also implicated in mammalian and *Drosophila* aging phenotypes.

Regulated apoptosis is required for normal homeostasis in dividing tissues such as the gut and hematopoetic system, and abnormal apoptotic events have been observed in muscle and other tissues during mammalian aging [6].  In addition, apoptosis is implicated in several human aging-related diseases, for example neurodegenerative diseases such as Alzheimer's disease and Parkinson's disease [7].  In aging *Drosophila*, abnormal apoptotic events have been observed in muscle and fat tissue [8], but the extent to which apoptosis (or cellular senescence) might modulate *Drosophila* life span remains largely unknown. Several genes that can affect apoptosis (and senescence) have been found to affect *Drosophila* life span, including *DPOSH*, *MnSOD* and *p53* [9-12]. In mammals hyperactive *p53* can produce an accelerated-aging-like phenotype [13], and in *Drosophila* a dominant-mutant *p53* transgene can inhibit insulin-like signaling and cause increased life span [14]. However, the extent to which these effects on life span might be mediated by alterations in apoptosis and/or cellular senescence pathways is largely unknown.  The potential importance of the cellular senescence and apoptosis pathways in modulating life span prompted a screen of additional genes implicated in these pathways for life span effects in the fly.

Conditional gene expression systems have several advantages for studies of aging: for example with the Tet-on system the expression of transgenes is triggered by feeding the flies the drug doxycycline, and with the Geneswitch system transgene expression is triggered using the drug RU486/Mifepristone [15-17]. These conditional systems allow for transgene expression to be limited to specific life cycle stages such as development or adulthood.  Moreover, these systems provide powerful controls for genetic background effects on life span, since the control and gene-over-expressing animals have identical genetic backgrounds and differ only in the presence or absence of the drug.  It is often desirable to over-express a gene in all the tissues of the fly, for example when screening genes for possible life span effects.  We have recently reported the generation of a Geneswitch system driver (called "Act-GS-255B"), which contains multiple inserts of a construct in which the promoter of the cytoplasmic actin gene *Actin5C* is used to drive expression of the Geneswitch transcription factor [16].  Here the Act-GS-255B driver is further characterized using a UAS-GFP reporter, and we report that it is truly tissue-general in both the larval and adult stages. The tissue-general driver facilitated the screening of senescence and apoptosis regulatory genes for life span effects.

## Results

### Characterization of Geneswitch drivers in adult flies using the UAS-GFP reporter

**Figure 1.**
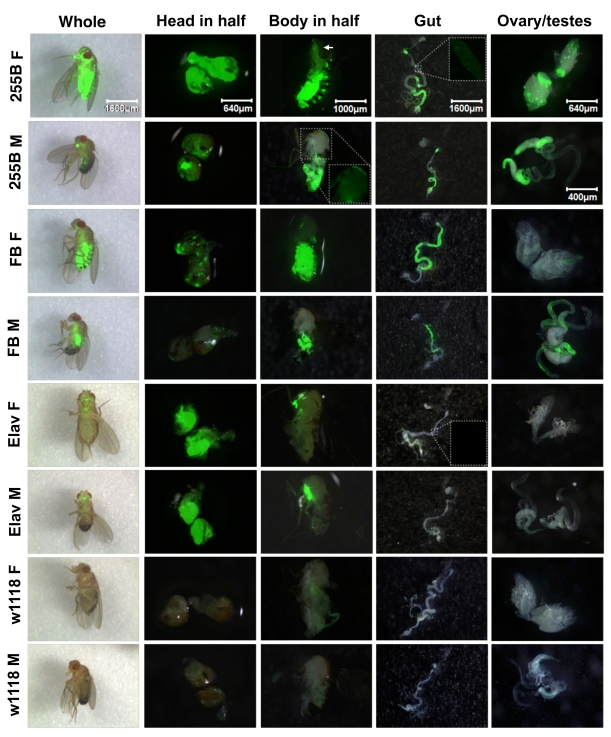
Expression pattern produced by Geneswitch drivers and UAS-GFP reporter in adult flies. The indicated GeneSwitch drivers Act-GS-255B ("255B"), Elav-GS ("Elav") and WB-FB-GS ("FB") were crossed to the UAS-ultraGFP reporter and adult progeny containing both constructs were scored for GFP expression in various tissues.  Control flies were generated by crossing UAS-ultraGFP to *white^1118^*strain flies to produce progeny containing only UAS-ultraGFP.  Age-synchronized flies were cultured in the presence and absence of the drug RU486 for two weeks prior to assay, and GFP expression was scored in whole adult flies and dissected tissues, as indicated.  Each image is the overlay of the visible light and GFP images.  Insets show details of the regions boxed in white, GFP image only.  M = male, F = female.  Pictures were taken at the magnification of 20X, 50X, 32X, 20X, 50X, and 80X, for whole fly, head in half, body in half, gut, ovary, and testes, respectively. The white arrow indicates a region of 255B Female flight muscle that is obscured by a fragment of cuticle.

To facilitate the screen of apoptosis and senescence-regulatory genes for life span effects, several Geneswitch system drivers were characterized for their tissue-specificity of transgene activation using a UAS-GFP reporter, both in adult flies and during larval development.  The UAS-GFP reporter employed was "UAS-ultraGFP" which contains multiple copies of a UAS-eGFP construct, and yields particularly high levels of GFP expression [18].   Three Geneswitch system drivers were characterized: The Act-GS-255B driver strain contains multiple inserts of a construct in which the promoter from the cytoplasmic actin gene *Actin 5C* drives Geneswitch, and is expected to yield tissue-general expression [16]. The Elav-GS driver contains Geneswitch under control of the *Elav* gene promoter and produces nervous-system-specific expression [19].  Finally the whole-body fat-body Geneswitch driver strain ("WB-FB-GS") contains both a head fat-body driver (S_1_-32) and a body-fat-body driver (S_1_-106) [20-22], and is expected to yield expression in the fat-body tissue throughout the animal. The three driver strains were crossed to the UAS-ultraGFP reporter strain to produce adult progeny containing both the driver and reporter constructs, and the flies were cultured in the presence and absence of drug for two weeks.   GFP expression was scored in live adult flies as well as in several dissected tissues (Figure 1).  The Act-GS-255B driver was found to yield tissue-general expression of the UAS-ultraGFP reporter in adult flies.  In whole adults, GFP expression was observed throughout the body of both males and females, with greater expression levels observed in females relative to males.  Similarly with heads dissected in half and bodies dissected in half, expression was observed in all tissues, including abundant expression in nervous system, muscle (including flight muscle), and fat-body tissue.  Note that flight muscle in male has lower expression than flight muscle in female, however inspection of the GFP-only image for male flight muscle (inset) reveals expression throughout this tissue.  Abundant expression was also observed throughout dissected gut tissue, ovary and testes.  The expression level was greater in some regions of the gut than others, however all regions of the gut exhibited staining, as revealed by inspection of the GFP-only images (inset). All tissues observed showed significant GFP expression, and therefore we conclude that Act-GS-255B yields truly tissue-general expression in  adult flies.  The  WB-FB-GS  driver produced  GFP expression in the head-fat-body and body-fat-body tissues, as expected, as well as in the gut and testes, and very faint expression in ovary; there was no detectable expression in nervous, muscle, or other tissues.  Notably, the expression in adult male head fat body was much reduced relative to female head fat body, consistent with recent characterization of the fat body drivers using a LacZ reporter  [17].   Finally,  the  Elav-GS driver produced  abundant  expression  in  the brain and ventral nerve cord, as expected, and there was no detectable expression in any other tissues; for example, the muscle, gut and gonads were clearly negative. Note the GFP-only image for the gut (inset) shows a lack of expression.  The Elav-GS driver was found to produce similar levels of UAS-GFP reporter expression in male versus female in our experiments.

### Characterization of Geneswitch drivers in larvae using the UAS-GFP reporter

**Figure 2.**
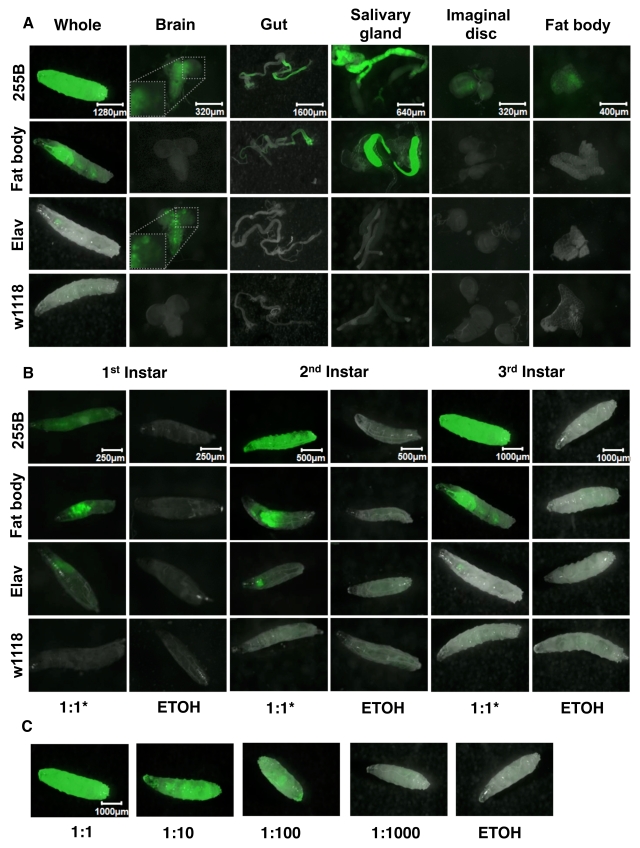
Expression pattern produced by GeneSwitch drivers and UAS-GFP reporter in larvae. The crosses are the same as Figure 1, but larvae were cultured in the presence and absence of drug in the food, from hatching to the indicated developmental stage.  A. Expression patterns in 3^rd^ instar larvae and dissected tissues. For the Elav-GS driver ("Elav") a 1:10 dilution of drug was used because of the toxic effects of drug observed in larvae with this driver. Pictures were taken at the magnification of 25X, 100X, 20X, 50X, 100X, 80X, for whole larvae, brain, gut, salivary gland, imaginal discs, and fat body, respectively.  B. Expression patterns in the three larval stages. For Elav-GS a 1:10 dilution of drug was used to avoid toxic effects. GFP pictures were taken at the magnification of 100X, 50X, 25X, for 1^st^ instar, 2^nd^ instar, and 3^rd^ instar, respectively. C. Expression in 3^rd^ instar larvae using Act-GS-255B and titrations of drug. ETOH indicates the ethanol solvent for the drug alone.  Pictures were taken at the magnification of 25X.

The Geneswitch driver strains were also scored for expression patterns in 3^rd^ instar larvae and dissected tissues (Figure 2).  The Act-GS-255B  driver was  found to yield tissue-general expression, including abundant expression throughout the body of whole 3^rd^ instar larvae, as well as in dissected brain, gut, salivary gland, imaginal discs and fat-body tissues; all tissues observed showed abundant GFP expression (Figure 2A).  The inset for the Act-GS-255B 3^rd^ instar larval brain shows detail of the GFP-only image, and indicates that expression was present throughout the brain, with higher-level expression in a subset of cells.  The WB-FB-GS driver was found to drive abundant expression in salivary gland and anterior midgut, but notably no expression in any other larval tissues including larval fat-body. Finally the Elav-GS driver produced abundant expression in larval nervous system and no detectable expression in any other larval tissues. The inset for the Elav-GS 3^rd^ instar larval brain shows detail  of  the  GFP-only  image, and shows that expression was present throughout the brain, with higher-level expression in a subset of cells. Notably this subset of cells was different from that observed above with Act-GS-255B.  Each of the three drivers was found to produce similar patterns of expression in 1^st^ and 2^nd^  instar larvae as well (Figure 1B).  When the Act-GS-255B driver was induced using dilutions of RU486 drug in the culture media, it produced a dose-response of GFP expression in 3^rd^ instar larvae (Figure 1D), as well as in adult flies (data not shown).

### Effect of apoptosis and senescence-regulatory gene over-expression on life span

Fourteen apoptosis and senescence regulatory genes were chosen for analysis based on their relevance to human apoptosis and senescence pathways and the availability of reagents for *Drosophila*. *Ras85D* is a *Drosophila* homolog of the human oncogene *Ras* that encodes a GTPase involved in signal transduction.

*Ras85D activated form* contains an amino acid substitution that causes Ras to be constitutively active [23], and *Ras85D dominant negative* (*DN*) form contains an amino acid substitution that causes it to inhibit the endogenous Ras protein [23,24].  Wingless is a *Drosophila* homolog of the human Wnt signaling protein involved in development and tumorigenesis [25].  Pk61C is a serine/threonine protein kinase related to human PDK-1 and involved in growth signaling [26].  DIAP1 is a *Drosophila* member of the inhibitor of apoptosis protein (IAP) family [27]. *Baculovirus p35* is a caspase inhibitor protein also related to the IAPs.  *Nemo* (*nmo*) is the *Drosophila* homolog of a human protein kinase regulatory subunit involved in NF-kappaB signaling pathway [28].  *Egfr* is the *Drosophila* homolog of the human epidermal growth factor receptor [29]. The *Drosophilapointed* (*pnt*) gene encodes a transcription factor homologous to human *Ets1* that is involved in the Ras signaling pathway.  The *DrosophilaMatrix metalloproteinase 2* gene (*Mmp2*) is involved in tissue remodeling  and tumor progression and is related to a family of human matrix metalloproteinases [30]. The *Drosophila* *Stat92E* gene encodes a homolog of the human Stat transcription factor, which is a target of the Jak-Stat growth-regulatory pathway [31].  The *Drosophilapuckered* (*puc*) gene encodes a phosphatase homologous to the human VH-1 family that antagonizes JNK signaling, and heterozygous *puc* mutant flies have been reported to have increased stress resistance and life span [32,33]. The *DrosophilaSphingosine kinase 2* (*Sk2*) gene encodes a lipid kinase involved in activation of protein kinase C-family signaling, and the human homolog *Sphk2* is implicated in regulation of apoptosis [34]. Finally the *CG14544* gene encodes a predicted methyltransferase, and the *Drosophilabantam* (*ban*) gene encodes a micro-RNA that inhibits expression of pro-apoptotic genes [35]. Each of these genes of interest was over-expressed in adult flies or during larval development, and assayed for effects on adult fly life span.

**Figure 3.**
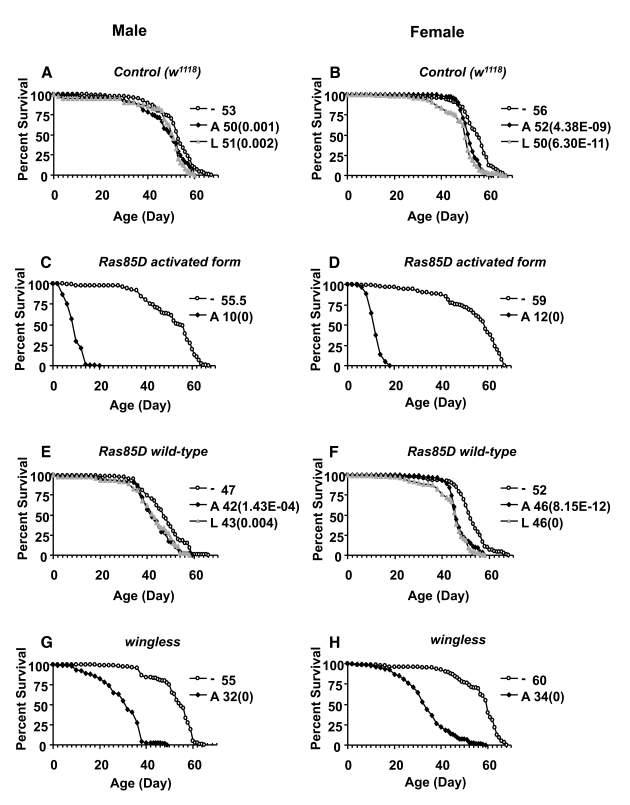
Effect of transgene over-expression on survival of adult flies. Apoptosis and senescence-related genes *wingless*, *Ras85D*, and *Ras85D activated form* were over-expressed during larval development or in adults, and assayed for effects on adult life span in male and female flies, as indicated. The life span assays were performed at 29°C. Open circles represent the no-drug control ("-"). Solid squares represent adults treated with drug ("A"). Grey triangles represent larvae on drug ("L"). Survival curves are plotted as a function of adult age in days. Median life span of each cohort is presented along with *p* value for log rank test  (in parentheses).  (**A, C, E, G**) male flies.  (**B, D, F, H**) female flies.  (**A, B**) Control flies containing the driver and no target transgene. (**C, D**) *Ras85D activated form*.  (**E, F**) *Ras85D* wild-type. (**G, H**) *wingless*.

**Figure 4.**
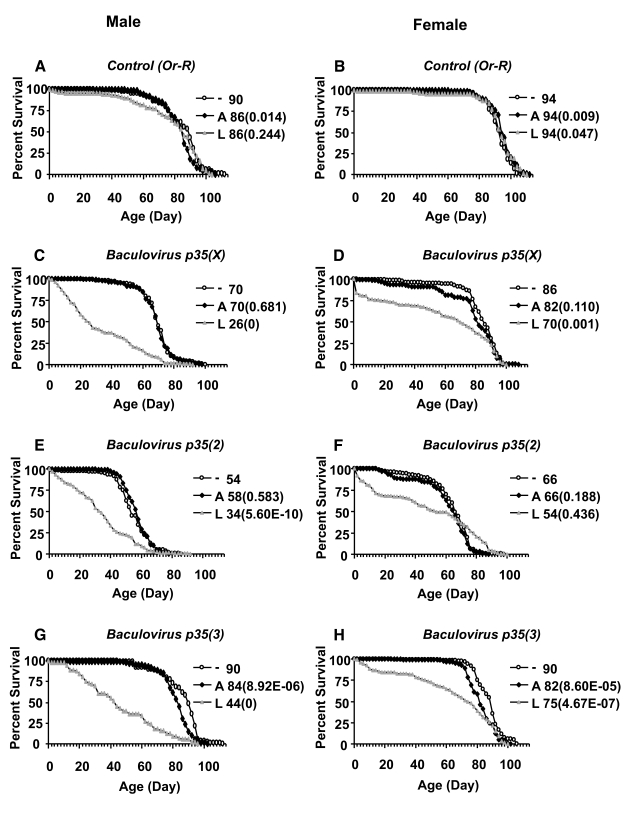
Effect of *Baculovirus p35* over-expression on survival of adult flies. *Baculovirus p35* transgenes inserted on the X chromosome, chromosome 2, and chromosome 3 were over-expressed during larval development or adult stage, as indicated. The life span assays were performed at 25°C. Open circles represent the no-drug control ("-"). Solid squares represent adults treated with drug ("A"). Grey triangles represent larvae on drug ("L"). Survival curves are plotted as a function of adult age in days. Median life span of each cohort is presented along with *p* value for log rank tests  (in parentheses). (**A, C, E, G**) male flies.  (**B, D, F, H**) female flies.  (**A,B**) Control flies containing the driver and no target transgene. (**C, D**) *Baculovirus p35* transgene on X chromosome.  (**E, F**) *Baculovirus p35* transgene on second chromosome. (**G, H**) *Baculovirus p35* transgene on third chromosome.

To control for any possible effects of the Geneswitch system and the RU486 drug itself, life span was assayed in flies that were the progeny of Act-GS-2555B driver crossed to either Oregon-R (Or-R) wild-type strain or to the *w^1118^* control strain, to produce progeny containing only the driver.  In these control flies, treatment with drug produced small, but statistically significant reductions in life span in both male and female adults: treatment during adulthood reduced mean life span by -4% to -10%, while treatment in larval stages reduced adult life span by -8% to -16% (Figure 3A, B; Figure 4A, B; Tables 2, 3).  There were no significant increases in life span in control flies treated with RU486 in any of the replicate experiments.  These data indicate that in these experiments, when the Act-GS-255B driver is present, the RU486 can cause small but significant reductions in adult life span, and this effect must be taken into account when interpreting the effects of transgene over-expression. Other studies [22], including ones from our own laboratory using the Act-GS-255B driver [36], found no negative effects of RU486 on adult fly life span. We conclude that the small negative effects observed here result from differences in the lot of RU486 drug, and/or small differences in effective concentrations due to specifics of media preparation. To confirm that the Act-GS-255B driver can produce increased life span, it was used to drive over-expression of the dominant *p53* allele (*p53-259H*).  Over-expression of *p53-259H* in adult flies using the ubiquitous Act-GS-255B driver produced increased median life span in females (+8%) but not males (-2.8%), and no life span increase when expressed in larvae (Table 3). These results are consistent with previous studies showing that expression of *p53-259H *in the adult nervous system with the Elav-GS driver can cause increased life span in females [14], and confirms that the Act-GS-255B driver can indeed produce increased life span when combined with an appropriate target gene.

Most of the genes tested by over-expression with the ubiquitous Act-GS-255B driver did not affect life span to an extent greater than the small changes observed with the control flies.  However, *Ras activated form* transgene was lethal when expressed in larvae, and reduced both male and female life span by -80% when expressed in adults (Figure 3C, D; Table 2).  Over-expression of wild-type *Ras* or a *Ras dominant-negative* allele was not lethal to larvae, and produced only small decreases (-4% to -12%) in both male and female adult life span (Figure 2 E, F; Table 2), thereby in the range of negative effects observed with control flies.  Over-expression of the *wingless* gene was found to be lethal to male and female larvae, using two independent *wingless* transgenes (Table 2).  Over-expression of *wingless* in adult flies produced significant reductions in both male and female life span: ~-42% with one *wingless* transgene (Figure 3 G, H) and ~-10% with the other transgene (Table 2).

**Figure 5.**
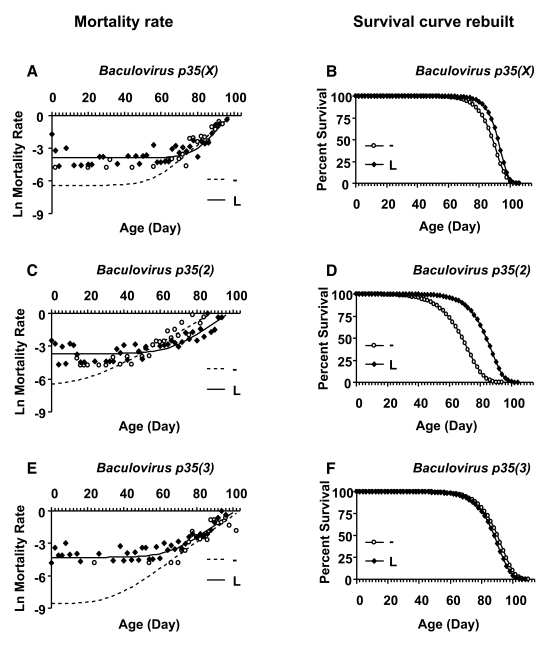
Mortality rate analysis of female larvae with and without *Baculovirus p35* transgene expression. Open circles represent the no-drug control ("-"). Solid squares represent larvae cultured with drug ("L"). (**A, B**) *Baculovirus p35* transgene on X chromosome. (**C, D**) *Baculovirusp35* transgeneon second chromosome. (**E, F**) *Baculovirus p35* transgene on third chromosome.  (**A, C, E**) Plots of natural-log mortality rate vs. age in days.  (**B, D, F**)  The data were fitted to the Gompertz-Makeham model, which best described the mortality rate. The age-independent mortality was removed and the survival curves were re-drawn using only the Gompertz components. Mortality rate analysis showed that age-independent mortality was significantly higher for female larvae on drug versus control for all three *Baculovirus p35* lines (Table 4).

Finally, the tissue-general Act-GS-255B driver was used to over-express three different transgenes encoding the caspase inhibitor *Baculovirus p35,* during larval development and in adult flies (Figure 4; Table 3).  Over-expression of *Baculovirusp35* in adult flies using the tissue-general Act-GS-255B driver produced only small decreases in life span that were within the range observed with control flies, suggesting there were no significant effects in adults.  In contrast, when *Baculovirus p35* was over-expressed during larval development using the tissue-general driver, it reduced the mean life span of male and female adults by -20% to -50%.  Interestingly, over-expression of each of the three independent *Baculovirus p35* transgenes during larval development produced an unusual biphasic-shaped survival curve in adult females (Figure 4 D, F, H), suggesting the presence of a subset of adult female flies  with  unchanged or  even  increased  life  span.  A Gompertz-Makeham model was found to give the best fit to the life span data for females in which *Baculovirus p35* was over-expressed during larval development (Figure 5; Table 4).  This analysis revealed that the decrease in mean life span was due to increased age-independent mortality. When the age-independent mortality was removed and the data re-plotted, it revealed a subset of female flies with unchanged (Figure 5 B, F) or increased life span (Figure 5D).

Two independent *Baculovirus p35* transgenes were also over-expressed in adult flies using the head-fat-body driver S_1_-32, and the whole-body fat-body driver (S_1_-32 plus S_1_-106), and during larval development using the whole-body fat-body driver, however no consistent effects on life span were observed (Table 3).

The nervous system-specific Elav-GS driver was also used to over-express two *baculovirus p35* transgenes.  In adults the Elav-GS driver itself had little to no effect on life span, and over-expression of *baculovirus p35* in adults using Elav-GS had no consistent effects on life span (Table 3).  In contrast, when drug was administered to larvae, the Elav-GS driver itself was associated with significant decreases in life span in both males (~-30% to -40%) and females (~-25%), and significantly reduced the number of male adults, and no effects of the *baculovirus p35* transgenes on life span could be identified in this background (Table 3).  In an attempt to reduce this background toxicity and allow assay of *baculovirus p35* transgenes with the Elav-GS driver in larvae, a 1:10 dilution of drug was used.  Under these conditions the life span reductions caused by drug in males and females were smaller (~-2% to -12%), and the number of males obtained was approximately normal, however no increases in life span were observed upon over-expression of *baculovirus p35* (Table 3).

The muscle-specific MHC-GS driver was used to drive over-expression of several transgenes in adult flies, however the MHC-GS driver itself was found to cause a significant RU486-dependent decrease in life span in both males and females (~-20% to -30%), and none of the target transgenes tested produced a significant life span increase in this background (Table 3).

**Table 1.** Starting Stocks

***St#***	***Genotype***	***Notes***	***Abbreviation***
*1*	*w; GS-Actin255-B:+*	*Ubiquitous GeneSwitch 255B Driver*	*255B*
*2*	*w; GS-Actin255-A;+*	*Ubiquitous GeneSwitch 255A Driver*	*255A*
*3*	*w; P{Switch}bun[Switch 1-32];+*	*GeneSwitch Head Fat Body Driver*	*S32*
*4*	*w; P{Switch}S1-106 P{Switch}bun[Switch 1-32];+*	*GeneSwitch Head & Thorax-Abdomen Fat Body Driver*	*S106 S32*
*5*	*yw; +; GS-Elav*	*GeneSwitch Elav Driver*	*Elav*
*6*	*yw; Sp/CyO,FLP.lacZ; MHC:GS*	*GeneSwitch Muscle Driver*	*Sp/CyO, MHC*
*7*	*Oregon R ( +; +; +)*	*wild type*	
*8*	*w1118; +; + *	*wild type*	
*9*	P{UAS.p35.H}BH3,w*;+;+	*UAS-p35 on chromosome 1*	*p35*
*10*	w*; P{UAS.p35.H}BH1;+	*UAS-p35 on chromosome 2*	*p35*
*11*	w*; +; P{UAS.p35.H}BH2	*UAS-p35 on chromosome 3*	*p35*
*12*	*w1118; +; P{UAS-Ras85D.V12}TL1*	*UAS-Ras85D activated form*	*Ras act*
*13*	w*; P{UAS-Ras85D.K}5-1;+	*UAS-Ras85D WT form*	*Ras WT*
*14*	*P{UAS-Ras85D.N17}TL1, w1118; +; +*	*UAS-Ras85D DN form*	*Ras DN*
*15*	w*; P{UAS-wg.H.T:HA1}3C;+	*UAS-wg on chromosome 2*	*wg^a^*
*16*	w*; +; P{UAS-wg.H.T:HA1}6C	*UAS-wg on chromosome 3*	*wg^b^*
*17*	*y1 w67c23; +;P{EPgy2}EY04093*	*EP-Pk61C*	*Pk61C^a^*
*18*	*w; +; P{EP}Pk61CEP3644/TM6,Tb*	*EP-Pk61C*	*Pk61C^b^*
*19*	w*; +; P{UAS-DIAP1.H}3	*UAS-DIAP1*	*DIAP1*
*20*	*y1 w67c23; P{EPgy2}EY00935*	*EP-nmo*	*nmo*
*21*	y1 w*; +; P{UAS-Egfr.B}32-26-1	*UAS-Egfr*	*Egfr*
*22*	*y1 w67c23; +; P{EPgy2}pntEY03254*	*EP-pnt*	*pnt*
*23*	*y1 w67c23; P{EPgy2}Mmp2EY08942/CyO; +*	*EP-Mmp2*	*Mmp2*
*24*	*y1 w67c23; +; P{EPgy2}Stat92EEY14209/TM3, Sb1 Ser1*	*EP-Stat92E*	*Stat*
*25*	w*; +; P{EPgy2}pucEY09772/TM6C	*EP-puc*	*puc*
*26*	*y1 w67c23; +; P{EPgy2}scramb2EY01180*	*EP-Sk2*	*Sk2*
*27*	*y1 w67c23; +; P{EPgy2}EY06207*	*EP-ban*	*ban*
*28*	*w1118; +; PBac{WH}CG14544f01091/TM6B, Tb1*	*XP-CG14544*	*CG14544*
*29*	*w1118; +; P{GUS-p53.259H}3.1*	*UAS-p53 point mutation *	*p53.259H*

**Table 2.** Life span data of apoptosis-related gene experiments, with means, standard deviations, medians, percent change in mean and median, and log rank p value.

**CrossMxF**	**RU486**	***Genotype***	**Sex**	**N**	**Mean*^a^***	**Median**	**%Change in Mean**	**%Change in Median**	**Log Rank p Value **
*Exp1 Life span assay using GS255B driver at 29C *									
8-1	-	*w/Y; 255B/+; +*	M	115	51.53±8.66	53	---------	---------	---------
	A	*w/Y; 255B/+; +*	M	120	47.29±11.06	50	-8.23	-5.66	0.001
	L	*w/Y; 255B/+; +*	M	40	47.28±12.15	51	-8.26	-3.77	0.002
	-	*w/w; 255B/+; +*	F	128	54.18±8.26	56	---------	---------	---------
	A	*w/w; 255B/+; +*	F	120	51.74±3.89	52	-4.5	-7.14	4.38E-09
	L	*w/w; 255B/+; +*	F	120	48.18±8.38	50	-11.07	-10.71	6.30E-11
12-1	-	*w/Y; 255B/+; Ras act/+*	M	122	51.11±11.58	55.5	---------	---------	---------
	A	*w/Y; 255B/+; Ras act/+*	M	128	9.45±3.42	10	-81.5	-81.98	0
	L	*w/Y; 255B/+; Ras act/+*	M	0	NA	NA	---------	---------	---------
	-	w/w*; 255B/+; Ras act/+	F	123	54.19±13.26	59	---------	---------	---------
	A	w/w*; 255B/+; Ras act/+	F	123	12.11±2.8	12	-77.64	-79.66	0
	L	w/w*; 255B/+; Ras act/+	F	0	NA	NA	---------	---------	---------
13-1	-	*w/Y; 255B/Ras WT;+*	M	124	46.65±9.01	47	---------	---------	---------
	A	*w/Y; 255B/Ras WT;+*	M	122	42.3±9.13	42	-9.32	-10.64	1.43E-04
	L	*w/Y; 255B/Ras WT;+*	M	47	42.06±10.37	43	-9.84	-8.51	0.004
	-	w/w*; 255B/Ras WT;+	F	126	51.31±8.64	52	---------	---------	---------
	A	w/w*; 255B/Ras WT;+	F	126	46.84±5.17	46	-8.71	-11.54	8.15E-12
	L	w/w*; 255B/Ras WT;+	F	118	43.66±8.85	46	-14.91	-11.54	0
1-14	-	*Ras DN, w/Y; 255B/+; +*	M	127	47.89±9.88	50	---------	---------	---------
	A	*Ras DN, w/Y; 255B/+; +*	M	123	43.64±6.87	44	-8.87	-12	5.78E-08
	L	*Ras DN, w/Y; 255B/+; +*	M	79	44.76±12.49	48	-6.54	-4	0.14
	-	*Ras DN, w/w; 255B/+; +*	F	121	51.65±14.25	57	---------	---------	---------
	A	*Ras DN, w/w; 255B/+; +*	F	125	51.82±8.3	53	0.32	-7.02	5.09E-04
	L	*Ras DN, w/w; 255B/+; +*	F	125	45.39±13.16	49	-12.12	-14.04	1.98E-09
15-1	-	*w/Y; 255B/wg^a^; +*	M	130	52.56±8.37	55	---------	---------	---------
	A	*w/Y; 255B/wg^a^; +*	M	122	29.83±9.32	32	-43.25	-41.82	0
	L	*w/Y; 255B/wg^a^; +*	M	0	NA	NA	---------	---------	---------
	-	w/w*; 255B/wg^a^; +	F	122	55.78±12.01	60	---------	---------	---------
	A	w/w*; 255B/wg^a^; +	F	125	33.61±10.55	34	-39.75	-43.33	0
	L	w/w*; 255B/wg^a^; +	F	0	NA	NA	---------	---------	---------
16-1	-	*w/Y; 255B/+;wg^b^/+*	M	124	52.31±8.81	56	---------	---------	---------
	A	*w/Y; 255B/+;wg^b^/+*	M	131	45.02±8.04	47	-13.94	-16.07	0
	L	*w/Y; 255B/+;wg^b^/+*	M	0	NA	NA	---------	---------	---------
	-	w/w*; 255B/+;wg^b^/+	F	120	51.29±10.38	53	---------	---------	---------
	A	w/w*; 255B/+;wg^b^/+	F	123	47.55±7.23	49	-7.29	-7.55	3.24E-10
	L	w/w*; 255B/+;wg^b^/+	F	0	NA	NA	---------	---------	---------
17-1	-	*w/Y; 255B/+; Pk61C^a^/+*	M	122	47.9±9.67	47	---------	---------	---------
	L	*w/Y; 255B/+; Pk61C^a^/+*	M	21	42.81±13.89	47	-10.63	0	0.224
	-	*w/yw; 255B/+; Pk61C^a^/+*	F	127	49.82±16.97	56	---------	---------	---------
	L	*w/yw; 255B/+; Pk61C^a^/+*	F	121	50.24±11.55	52	0.84	-7.14	8.57E-04
18-1	-	*w/Y; 255B/+; Pk61C^b^/+*	M	126	57.29±8.23	59	---------	---------	---------
	L	*w/Y; 255B/+; Pk61C^b^/+*	M	24	48.08±10.99	51	-16.06	-13.56	4.11E-12
	-	*w; 255B/+; Pk61C^b^/+*	F	124	54.51±9.89	56.5	---------	---------	---------
	L	*w; 255B/+; Pk61C^b^/+*	F	121	45.28±13.38	51	-16.93	-9.73	1.79E-14
19-1	-	*w/Y; 255B/+; DIAP1/+*	M	120	56.23±8.68	59	---------	---------	---------
	L	*w/Y; 255B/+; DIAP1/+*	M	93	47.68±17.07	53	-15.22	-10.17	0.002
	-	w/w*; 255B/+; DIAP1/+	F	120	54.64±11.2	57.5	---------	---------	---------
	L	w/w*; 255B/+; DIAP1/+	F	117	46.74±14.76	51	-14.45	-11.3	1.97E-07
7-1	-	*w/Y; 255B/+; +*	M	124	52.97±7.68	56	---------	---------	---------
	A	*w/Y; 255B/+; +*	M	124	49.73±4.6	50	-6.11	-10.71	6.19E-13
	L	*w/Y; 255B/+; +*	M	22	44.5±16.86	52	-15.99	-7.14	2.94E-05
	-	*w/+; 255B/+; +*	F	118	58.39±5.25	59	---------	---------	---------
	A	*w/+; 255B/+; +*	F	122	52.66±4.09	52	-9.81	-11.86	0
	L	*w/+; 255B/+; +*	F	122	50.45±9.78	53.5	-13.6	-9.32	1.18E-13
*Ex 2 Life span assay using GS255B driver at 25C *									
7-1	-	*w/Y; 255B/+; +*	M	94	73.17±15.64	78	---------	---------	---------
	A	*w/Y; 255B/+; +*	M	93	69.97±12.33	72	-4.38	-7.69	5.22E-04
	-	*w/+; 255B/+; +*	F	92	87.2±18.44	92	---------	---------	---------
	A	*w/+; 255B/+; +*	F	91	91.93±7.76	94	5.43	2.17	0.940
20-1	-	*w/Y; 255B/+; nmo/+*	M	95	66.74±16.11	68	---------	---------	---------
	A	*w/Y; 255B/+; nmo/+*	M	90	64.66±14.3	66	-3.12	-2.94	0.102
	-	*w/yw; 255B/+; nmo/+*	F	97	67.59±28.66	74	---------	---------	---------
	A	*w/yw; 255B/+; nmo/+*	F	95	68.79±30.17	80	1.78	8.11	0.878
15-1	-	*w/Y; 255B/wg^a^; +*	M	96	72.88±10.31	74	---------	---------	---------
	A	*w/Y; 255B/wg^a^; +*	M	92	53.14±18.01	56	-27.08	-24.32	0
	-	w/w*; 255B/wg^a^; +	F	97	78.89±19.38	84	---------	---------	---------
	A	w/w*; 255B/wg^a^; +	F	97	53.72±22.13	52	-31.9	-38.1	0
17-1	-	*w/Y; 255B/+; Pk61C^a^/+*	M	91	64.11±13.4	64	---------	---------	---------
	A	*w/Y; 255B/+; Pk61C^a^/+*	M	94	62.85±13.08	66	-1.96	3.13	0.555
	-	*w/yw; 255B/+; Pk61C^a^/+*	F	98	70.73±26.23	78	---------	---------	---------
	A	*w/yw; 255B/+; Pk61C^a^/+*	F	94	79.81±23.77	90	12.83	15.38	0.149
21-1	-	*w/Y; 255B/+; Egfr/+*	M	89	62.38±11.19	66	---------	---------	---------
	A	*w/Y; 255B/+; Egfr/+*	M	97	62.06±9.18	64	-0.51	-3.03	0.166
	-	w/y w*; 255B/+; Egfr/+	F	95	65.71±21.16	68	---------	---------	---------
	A	w/y w*; 255B/+; Egfr/+	F	100	63.52±17.9	65	-3.33	-4.41	0.076
19-1	-	*w/Y; 255B/+; DIAP1/+*	M	102	76.57±13.04	78	---------	---------	---------
	A	*w/Y; 255B/+; DIAP1/+*	M	94	73.4±9.53	74	-4.13	-5.13	0.002
	-	w/w*; 255B/+; DIAP1/+	F	98	78.9±18.26	84	---------	---------	---------
	A	w/w*; 255B/+; DIAP1/+	F	95	81.39±19.17	88	3.16	4.76	0.011
22-1	-	*w/Y; 255B/+; pnt/+*	M	96	62.6±9.74	64	---------	---------	---------
	A	*w/Y; 255B/+; pnt/+*	M	94	59.15±10.7	60	-5.52	-6.25	0.077
	-	*w/yw; 255B/+; pnt/+*	F	92	74.32±27.19	85	---------	---------	---------
	A	*w/yw; 255B/+; pnt/+*	F	95	79.77±20.03	88	7.34	3.53	0.402
*Exp3 Life span assay using GS255B driver at 25C *									
7-1	-	*w/Y; 255B/+; +*	M	100	81.01±15.38	86	---------	---------	---------
	A	*w/Y; 255B/+; +*	M	92	80.46±10.42	82	-0.68	-4.65	0.039
	-	*w/+; 255B/+; +*	F	85	92.49±11.86	94	---------	---------	---------
	A	*w/+; 255B/+; +*	F	99	92.05±13.07	94	-0.48	0	0.571
13-1	-	*w/Y; 255B/Ras WT;+*	M	95	75.87±12.26	78	---------	---------	---------
	A	*w/Y; 255B/Ras WT;+*	M	98	67.92±14.13	70	-10.48	-10.26	1.62E-06
	-	w/w*; 255B/Ras WT;+	F	96	83.43±12.26	86	---------	---------	---------
	A	w/w*; 255B/Ras WT;+	F	98	79.59±10.58	82	-4.6	-4.65	0.001
23-1	-	*w/Y; 255B/Mmp2; +*	M	96	69.77±13.03	70	---------	---------	---------
	A	*w/Y; 255B/Mmp2; +*	M	96	68.81±10.06	70	-1.37	0	0.117
	-	*w/yw; 255B/Mmp2; +*	F	98	85.94±18.45	91	---------	---------	---------
	A	*w/yw; 255B/Mmp2; +*	F	101	84.85±18.67	90	-1.27	-1.1	0.109
24-1	-	*w/Y; 255B/+; Stat/+*	M	96	64.31±10.06	65	---------	---------	---------
	A	*w/Y; 255B/+; Stat/+*	M	99	65.08±13.33	68	1.19	4.62	0.325
	-	*w/yw; 255B/+; Stat/+*	F	99	70.48±21.48	78	---------	---------	---------
	A	*w/yw; 255B/+; Stat/+*	F	96	62.29±24.99	74	-11.62	-5.13	0.076
25-1	-	*w/Y; 255B/+; puc/+*	M	97	70.78±14.98	70	---------	---------	---------
	A	*w/Y; 255B/+; puc/+*	M	96	68.96±13.62	68	-2.58	-2.86	0.269
	-	w/w*; 255B/+; puc/+	F	84	94.07±15.00	98	---------	---------	---------
	A	w/w*; 255B/+; puc/+	F	97	98.1±7.86	100	4.29	2.04	0.135
*Exp4 Life span assay using GS255B driver at 25C *									
7-1	-	*w/Y; 255B/+; +*	M	92	73.76±18.31	78	---------	---------	---------
	A	*w/Y; 255B/+; +*	M	86	71.84±10.87	74	-2.61	-5.13	1.43E-04
	-	*w/+; 255B/+; +*	F	86	86.28±15.46	90	---------	---------	---------
	A	*w/+; 255B/+; +*	F	101	86.18±10.02	88	-0.12	-2.22	0.035
26-1	-	*w/Y; 255B/+; Sk2/+*	M	90	67.22±16.97	72	---------	---------	---------
	A	*w/Y; 255B/+; Sk2/+*	M	95	69.85±12.26	72	3.91	0	0.953
	-	*w/yw; 255B/+; Sk2/+*	F	101	73.29±24.6	84	---------	---------	---------
	A	*w/yw; 255B/+; Sk2/+*	F	106	78.08±19.61	86	6.53	2.38	0.84
27-1	-	*w/Y; 255B/+; ban/+*	M	98	66.59±21.91	70	---------	---------	---------
	A	*w/Y; 255B/+; ban/+*	M	95	61.56±18.15	62	-7.56	-11.43	0.003
	-	*w/yw; 255B/+; ban/+*	F	94	76.36±28.78	88	---------	---------	---------
	A	*w/yw; 255B/+; ban/+*	F	96	81.56±18.03	88	6.81	0	0.023
28-1	-	*w/Y; 255B/+;CG14544/+*	M	91	75.03±12.8	76	---------	---------	---------
	A	*w/Y; 255B/+;CG14544/+*	M	97	77.01±9.82	78	2.64	2.63	0.844
	-	*w/w; 255B/+;CG14544/+*	F	101	70.99±26.75	82	---------	---------	---------
	A	*w/w; 255B/+;CG14544/+*	F	96	69.33±20.57	79	-2.33	-3.66	2.28E-05
*Exp5 Life span assay using GS255B driver, and MHC GS driver at 25C *									
7-1	-	*w/Y; 255B/+; +*	M	98	75.06±11.65	79	---------	---------	---------
	A	*w/Y; 255B/+; +*	M	97	73.96±12.27	78	-1.47	-1.27	0.161
	-	*w/+; 255B/+; +*	F	100	87.88±7.74	88	---------	---------	---------
	A	*w/+; 255B/+; +*	F	101	85.33±12.78	88	-2.91	0	0.014
8-1	-	*w/Y; 255B/+; +*	M	99	66.55±11.82	68	---------	---------	---------
	A	*w/Y; 255B/+; +*	M	97	69.84±10.57	72	4.94	5.88	0.14
	-	*w/w; 255B/+; +*	F	100	79.6±14.08	84	---------	---------	---------
	A	*w/w; 255B/+; +*	F	97	81.69±3.82	82	2.63	-2.38	0.005
17-1	-	*w/Y; 255B/+; Pk61C^a^/+*	M	99	64.87±12.41	64	---------	---------	---------
	A	*w/Y; 255B/+; Pk61C^a^/+*	M	98	62.16±12.33	64	-4.17	0	0.113
	-	*w/yw; 255B/+; Pk61C^a^/+*	F	98	81.96±13.91	86	---------	---------	---------
	A	*w/yw; 255B/+; Pk61C^a^/+*	F	99	80.46±11.15	82	-1.82	-4.65	0.001
18-1	-	*w/Y; 255B/+; Pk61C^b^/+*	M	100	71.2±9.32	74	---------	---------	---------
	A	*w/Y; 255B/+; Pk61C^b^/+*	M	101	73.33±8.19	74	2.99	0	0.135
	-	*w; 255B/+; Pk61C^b^/+*	F	98	80.27±12.63	82	---------	---------	---------
	A	*w; 255B/+; Pk61C^b^/+*	F	100	82.02±4.89	82	2.19	0	0.399
19-1	-	*w/Y; 255B/+; DIAP1/+*	M	101	72.97±11.13	76	---------	---------	---------
	A	*w/Y; 255B/+; DIAP1/+*	M	101	71.15±11.37	74	-2.5	-2.63	0.425
	-	w/w*; 255B/+; DIAP1/+	F	98	83.02±7.52	84	---------	---------	---------
	A	w/w*; 255B/+; DIAP1/+	F	106	79.47±12.78	82	-4.27	-2.38	0.011
7-6	-	*yw/Y;+/CyO; MHC/+*	M	96	71.31±13.18	76	---------	---------	---------
	A	*yw/Y;+/CyO; MHC/+*	M	98	58.92±12.84	60	-17.38	-21.05	0
	-	*yw/+;+/CyO; MHC/+*	F	96	78.04±15.76	84	---------	---------	---------
	A	*yw/+;+/CyO; MHC/+*	F	114	52.84±13.75	58	-32.29	-30.95	0
8-6	-	*yw/Y;+/CyO; MHC/+*	M	99	56.42±16.69	60	---------	---------	---------
	A	*yw/Y;+/CyO; MHC/+*	M	92	43.26±15	48	-23.33	-20	1.61E-12
	-	*yw/w;+/CyO; MHC/+*	F	93	60.73±18.82	68	---------	---------	---------
	A	*yw/w;+/CyO; MHC/+*	F	99	45.21±13.63	50	-25.55	-26.47	2.22E-16
17-6	-	*yw/Y;+/CyO; MHC/Pk61C^a^*	M	100	54.32±14.66	58	---------	---------	---------
	A	*yw/Y;+/CyO; MHC/Pk61C^a^*	M	101	52.46±12.42	56	-3.43	-3.45	0.004
	-	*yw;+/CyO; MHC/Pk61C^a^*	F	100	61.3±19.84	65	---------	---------	---------
	A	*yw;+/CyO; MHC/Pk61C^a^*	F	99	39.37±14.22	36	-35.77	-44.62	0
18-6	-	*yw/Y;+/CyO; MHC/Pk61C^b^*	M	96	56.44±18.89	62	---------	---------	---------
	A	*yw/Y;+/CyO; MHC/Pk61C^b^*	M	108	53.56±10.21	56	-5.11	-9.68	1.75E-07
	-	*yw/w;+/CyO; MHC/Pk61C^b^*	F	96	55.25±21.21	56	---------	---------	---------
	A	*yw/w;+/CyO; MHC/Pk61C^b^*	F	90	39.42±12.45	38	-28.65	-32.14	1.40E-11
19-6	-	*yw/Y;+/CyO; MHC/DIAP1*	M	98	66.71±12.27	68	---------	---------	---------
	A	*yw/Y;+/CyO; MHC/DIAP1*	M	98	54.92±10.4	56	-17.68	-17.65	6.66E-15
	-	yw/w*;+/CyO;MHC/DIAP1	F	108	64.89±18.19	71	---------	---------	---------
	A	yw/w*;+/CyO;MHC/DIAP1	F	104	57.04±14.85	58	-12.1	-18.31	6.72E-08
7-6	-	*yw/Y;+/Sp; MHC/+*	M	99	68.67±13.38	74	---------	---------	---------
	A	*yw/Y;+/Sp; MHC/+*	M	98	61.76±12.62	64	-10.07	-13.51	5.34E-08
	-	*yw/+;+/Sp; MHC/+*	F	94	73.66±13.99	78	---------	---------	---------
	A	*yw/+;+/Sp; MHC/+*	F	102	59.98±8.45	62	-18.57	-20.51	0
8-6	-	*yw/Y;+/Sp; MHC/+*	M	96	61.6±14.81	66	---------	---------	---------
	A	*yw/Y;+/Sp; MHC/+*	M	94	54.55±15.5	56	-11.45	-15.15	2.47E-06
	-	*yw/w;+/Sp; MHC/+*	F	93	58.41±17.34	62	---------	---------	---------
	A	*yw/w;+/Sp; MHC/+*	F	102	47.59±12.97	52	-18.53	-16.13	2.70E-14
17-6	-	*yw/Y;+/Sp; MHC/Pk61C^a^*	M	95	49.31±12.75	54	---------	---------	---------
	A	*yw/Y;+/Sp; MHC/Pk61C^a^*	M	100	45.14±11.03	48	-8.45	-11.11	1.95E-04
	-	*yw;+/Sp; MHC/Pk61C^a^*	F	99	42.99±20.17	40	---------	---------	---------
	A	*yw;+/Sp; MHC/Pk61C^a^*	F	100	35.78±16.13	30	-16.77	-25	0.003
18-6	-	*yw/Y;+/Sp; MHC/Pk61C^b^*	M	100	56.36±12.56	60	---------	---------	---------
	A	*yw/Y;+/Sp; MHC/Pk61C^b^*	M	98	51.06±9.77	52	-9.4	-13.33	2.13E-06
	-	*yw/w;+/Sp; MHC/Pk61C^b^*	F	97	56.6±16.25	60	---------	---------	---------
	A	*yw/w;+/Sp; MHC/Pk61C^b^*	F	94	41.79±11.81	42	-26.17	-30	1.67E-15
19-6	-	*yw/Y;+/Sp; MHC/DIAP1*	M	99	61.21±10.04	62	---------	---------	---------
	A	*yw/Y;+/Sp; MHC/DIAP1*	M	97	55.51±9.17	58	-9.32	-6.45	5.02E-08
	-	yw/w*;+/Sp; MHC/DIAP1	F	103	71.13±17.02	78	---------	---------	---------
	A	yw/w*;+/Sp; MHC/DIAP1	F	101	62.3±13.23	66	-12.41	-15.38	6.66E-16

**Table 3.** Life span data for baculovirus p35 experiments, with means, standard deviations, medians, percent change in mean and median, and log rank p value.

**CrossMxF**	**RU486**	***Genotype***	**Sex**	**N**	**Mean*^a^***	**Median**	**%Change in Mean**	**%Change in Median**	**Log Rank p Value **
*Exp1 Life span assay of three UAS-p35 lines and UAS-p53.259H with GS255B driver at 25C*									
7-1	-	*w/Y; 255B/+; +*	M	120	84.6±14.25	90	---------	---------	---------
	A	*w/Y; 255B/+; +*	M	119	83.08±10.94	86	-1.8	-4.44	0.014
	L	*w/Y; 255B/+; +*	M	123	78.44±22.48	86	-7.28	-4.44	0.244
	-	*w/+; 255B/+; +*	F	116	92.02±9.64	94	---------	---------	---------
	A	*w/+; 255B/+; +*	F	121	94.69±8.61	94	2.9	0	0.009
	L	*w/+; 255B/+; +*	F	124	91.97±15.74	94	-0.05	0	0.047
1-9	-	p35,w*/Y; 255B/+; +	M	120	68.93±12.49	70	---------	---------	---------
	A	p35,w*/Y; 255B/+; +	M	123	68.62±11.76	70	-0.45	0	0.681
	L	p35,w*/Y; 255B/+; +	M	98	33.1±22.91	26	-51.98	-62.86	0
	-	p35,w*/+; 255B/+; +	F	122	83.28±15.13	86	---------	---------	---------
	A	p35,w*/+; 255B/+; +	F	130	77.15±20.92	82	-7.36	-4.65	0.11
	L	p35,w*/+; 255B/+; +	F	125	57.52±35.8	70	-30.93	-18.6	0.001
10-1	-	*w/Y; 255B/p35; +*	M	117	54.48±13	54	---------	---------	---------
	A	*w/Y; 255B/p35; +*	M	121	57.26±8.45	58	5.1	7.41	0.583
	L	*w/Y; 255B/p35; +*	M	110	34.25±19.5	34	-37.13	-37.04	5.60E-10
	-	w/w*; 255B/p35; +	F	120	64.05±14.63	66	---------	---------	---------
	A	w/w*; 255B/p35; +	F	126	60.79±16.68	66	-5.09	0	0.188
	L	w/w*; 255B/p35; +	F	123	49.37±32.2	54	-22.92	-18.18	0.436
11-1	-	*w/Y; 255B/+; p35/+*	M	133	86.03±12.51	90	---------	---------	---------
	A	*w/Y; 255B/+; p35/+*	M	122	81.31±13.87	84	-5.49	-6.67	8.92E-06
	L	*w/Y; 255B/+; p35/+*	M	56	46.18±24.21	44	-46.32	-51.11	0
	-	w/w*; 255B/+; p35/+	F	126	87.54±10.04	90	---------	---------	---------
	A	w/w*; 255B/+; p35/+	F	127	82.19±9.91	82	-6.11	-8.89	8.60E-05
	L	w/w*; 255B/+; p35/+	F	126	64.63±29.62	75	-26.17	-16.67	4.67E-07
29-1	-	*w/Y; 255B/+;p53.259H/+*	M	118	71.54±13.86	72	---------	---------	---------
	A	*w/Y; 255B/+;p53.259H/+*	M	125	68.90±10.41	70	-3.70	-2.78	0.002
	L	*w/Y; 255B/+;p53.259H/+*	M	119	67.73±16.92	70	-5.33	-2.78	0.069
	-	*w; 255B/+; p53.259H/+*	F	119	75.40±8.50	76	---------	---------	---------
	A	*w; 255B/+; p53.259H/+*	F	119	80.66±10.98	82	6.98	7.89	4.05E-08
	L	*w; 255B/+; p53.259H/+*	F	125	70.24±22.02	76	-6.84	0	0.202
*Exp2 Life span assay of three UAS-p35 lines with head FB driver, whole body FB driver and GS255A driver at 25C*									
3-7	-	*+/Y; S32/+; +*	M	75	59.23±14.11	64	---------	---------	---------
	A	*+/Y; S32/+; +*	M	63	55.21±15.74	60	-6.79	-6.25	0.013
	-	*w/+; S32/+; +*	F	111	59.91±18.96	60	---------	---------	---------
	A	*w/+; S32/+; +*	F	115	63.77±17.71	66	6.45	10	0.263
3-9	-	p35,w*/Y; S32/+; +	M	122	62.69±10.62	64	---------	---------	---------
	A	p35,w*/Y; S32/+; +	M	105	58.3±13.45	60	-6.99	-6.25	0.022
	-	p35,w*/w; S32/+; +	F	112	59.95±25	72	---------	---------	---------
	A	p35,w*/w; S32/+; +	F	108	59±24.86	68	-1.58	-5.56	0.974
3-10	-	w*/Y; S32/p35; +	M	123	45.19±7.61	46	---------	---------	---------
	A	w*/Y; S32/p35; +	M	120	41.52±6.84	42	-8.12	-8.7	1.96E-04
	-	w*/w; S32/p35; +	F	121	61.62±8.71	62	---------	---------	---------
	A	w*/w; S32/p35; +	F	105	63.28±10.6	66	2.69	6.45	0.036
3-11	-	w*/Y; S32/+; p35/+	M	125	62.67±12.41	64	---------	---------	---------
	A	w*/Y; S32/+; p35/+	M	125	60.78±14.07	62	-3.03	-3.13	0.174
	-	w*/w; S32/+; p35/+	F	109	68.44±17.09	74	---------	---------	---------
	A	w*/w; S32/+; p35/+	F	113	70.52±18.12	76	3.04	2.7	0.043
4-7	-	*+/Y; S106 S32/+; +*	M	116	54.12±9.89	56	---------	---------	---------
	A	*+/Y; S106 S32/+; +*	M	118	52.68±9.77	52	-2.67	-7.14	0.208
	-	*w/+; S106 S32/+; +*	F	110	58.82±14.95	62	---------	---------	---------
	A	*w/+; S106 S32/+; +*	F	120	58.07±16.45	63	-1.28	1.61	0.569
4-9	-	p35,w*/Y; S106 S32/+; +	M	121	47.21±8.48	46	---------	---------	---------
	A	p35,w*/Y; S106 S32/+; +	M	110	47.67±10.28	48	0.99	4.35	0.263
	-	p35,w*/w; S106 S32/+; +	F	119	55.18±22.95	66	---------	---------	---------
	A	p35,w*/w; S106 S32/+; +	F	126	47.79±24.9	62	-13.38	-6.06	0.01
4-10	-	w*/Y; S106 S32/p35; +	M	125	33.39±4.44	34	---------	---------	---------
	A	w*/Y; S106 S32/p35; +	M	125	32.3±6.16	32	-3.26	-5.88	0.475
	-	w*/w; S106 S32/p35; +	F	121	49.55±8.14	50	---------	---------	---------
	A	w*/w; S106 S32/p35; +	F	121	50.84±8.85	50	2.6	0	0.107
4-11	-	w*/Y; S106 S32/+; p35/+	M	125	47.15±6.81	48	---------	---------	---------
	A	w*/Y; S106 S32/+; p35/+	M	117	48.6±8.42	48	3.07	0	0.072
	-	w*/w; S106 S32/+; p35/+	F	125	56.81±13.02	60	---------	---------	---------
	A	w*/w; S106 S32/+; p35/+	F	116	60.69±11.7	64	6.83	6.67	0.004
2-7	-	*+/Y; 255A/+; +*	M	114	66.04±8.95	67	---------	---------	---------
	A	*+/Y; 255A/+; +*	M	117	58.97±15.36	62	-10.69	-7.46	1.48E-05
	-	*w/+; 255A/+; +*	F	114	72.65±13.95	78	---------	---------	---------
	A	*w/+; 255A/+; +*	F	116	75.02±13.19	78	3.26	0	0.064
2-9	-	p35,w*/Y; 255A/+; +	M	111	65.98±14.65	66	---------	---------	---------
	A	p35,w*/Y; 255A/+; +	M	115	59.82±13.31	60	-9.34	-9.09	3.78E-05
	-	p35,w*/w; 255A/+; +	F	113	58.95±20.26	64	---------	---------	---------
	A	p35,w*/w; 255A/+; +	F	117	69.21±17.4	72	17.4	12.5	1.32E-06
2-10	-	w*/Y;255A/p35; +	M	113	48.98±9.74	48	---------	---------	---------
	A	w*/Y;255A/p35; +	M	125	47.66±7.19	48	-2.69	0	0.03
	-	w*/w; 255A/p35; +	F	115	60.57±16.71	66	---------	---------	---------
	A	w*/w; 255A/p35; +	F	118	62±17.79	70	2.35	6.06	0.052
2-11	-	w*/Y; 255A/+; p35/+	M	115	63.66±11.4	64	---------	---------	---------
	A	w*/Y; 255A/+; p35/+	M	114	64.92±9.41	64	1.98	0	0.776
	-	w*/w; 255A/+; p35/+	F	120	67.05±11.58	70	---------	---------	---------
	A	w*/w; 255A/+; p35/+	F	120	68.75±9.08	70	2.54	0	0.41
*Exp3 Life span assay of two UAS-p35 lines with whole body FB driver at 29C *									
7-4	-	*w/Y; S106 S32/+; +*	M	124	49.15±12.5	54	---------	---------	---------
	L	*w/Y; S106 S32/+; +*	M	121	49.11±10.75	52	-0.08	-3.7	0.655
	-	*w/+; S106 S32/+; +*	F	121	51.95±10.82	54	---------	---------	---------
	L	*w/+; S106 S32/+; +*	F	118	55.29±10.06	60	6.42	11.11	0.029
8-4	-	*w/Y; S106 S32/+; +*	M	121	47.16±10.27	48	---------	---------	---------
	L	*w/Y; S106 S32/+; +*	M	118	42.85±12.89	44	-9.14	-8.33	0.002
	-	*w/w; S106 S32/+; +*	F	124	50.48±11.91	56	---------	---------	---------
	L	*w/w; S106 S32/+; +*	F	125	51.63±8.47	54	2.27	-3.57	0.196
10-4	-	*w/Y; S106 S32/p35; +*	M	121	50.43±6.73	52	---------	---------	---------
	L	*w/Y; S106 S32/p35; +*	M	121	46.5±8.28	48	-7.8	-7.69	4.23E-05
	-	w*/w; S106 S32/p35; +	F	120	50.4±12.84	56	---------	---------	---------
	L	w*/w; S106 S32/p35; +	F	129	48.57±8.9	50	-3.62	-10.71	1.45E-05
11-4	-	*w/Y; S106 S32/+; p35/+*	M	126	44.03±6.5	46	---------	---------	---------
	L	*w/Y; S106 S32/+; p35/+*	M	122	41.92±10.42	46	-4.8	0	0.208
	-	w*/w; S106 S32/+; p35/+	F	122	58.03±6.59	60	---------	---------	---------
	L	w*/w; S106 S32/+; p35/+	F	124	54.81±8.05	56	-5.56	-6.67	8.84E-07
*Exp4 Life span assay of two UAS-p35 lines with Elav driver at 29C*									
7-5	-	*yw/Y; +/+; Elav/+*	M	131	53.92±7.15	54	---------	---------	---------
	A	*yw/Y; +/+; Elav/+*	M	129	52.33±8.14	53	-2.95	-1.85	0.083
	L	*yw/Y; +/+; Elav/+*	M	59	35.85±10.58	38	-33.51	-29.63	0
	-	*yw/+; +/+; Elav/+*	F	127	58.15±7.22	60	---------	---------	---------
	A	*yw/+; +/+; Elav/+*	F	129	57.11±5.19	58	-1.79	-3.33	0.013
	L	*yw/+; +/+; Elav/+*	F	120	43.46±7.94	44	-25.26	-26.67	0
8-5	-	*yw/Y; +/+; Elav/+*	M	126	44.08±8.36	44.5	---------	---------	---------
	A	*yw/Y; +/+; Elav/+*	M	120	43.32±8.76	45	-1.73	1.12	0.186
	L	*yw/Y; +/+; Elav/+*	M	102	26.24±8.51	26	-40.48	-41.57	0
	-	*yw/w; +/+; Elav/+*	F	124	46.88±9.92	50	---------	---------	---------
	A	*yw/w; +/+; Elav/+*	F	124	48.13±7.5	49.5	2.67	-1	0.406
	L	*yw/w; +/+; Elav/+*	F	114	34.82±10.34	36	-25.73	-28	0
10-5	-	*yw/Y; p35/+; Elav/+*	M	125	42.34±6.38	44	---------	---------	---------
	A	*yw/Y; p35/+; Elav/+*	M	122	43.34±10.34	46	2.38	4.55	0.007
	L	*yw/Y; p35/+; Elav/+*	M	9	20.89±10.3	26	-50.66	-40.91	0
	-	yw/w*; p35/+; Elav/+	F	121	49±10.63	52	---------	---------	---------
	A	yw/w*; p35/+; Elav/+	F	126	50.16±6.4	51	2.36	-1.92	0.014
	L	yw/w*; p35/+; Elav/+	F	9	28.22±10.27	32	-42.4	-38.46	1.60E-14
11-5	-	*yw/Y; +; Elav/p35*	M	120	51.24±10.46	54	---------	---------	---------
	A	*yw/Y; +; Elav/p35*	M	121	48.62±10.1	52	-5.12	-3.7	1.22E-06
	L	*yw/Y; +; Elav/p35*	M	1	10±NA	10	-80.48	-81.48	5.60E-10
	-	yw/w*; +; Elav/p35	F	118	56.77±3.89	58	---------	---------	---------
	A	yw/w*; +; Elav/p35	F	131	52.67±5.08	54	-7.22	-6.9	3.51E-13
	L	yw/w*; +; Elav/p35	F	0	NA	NA	---------	---------	---------
*Exp5 Life span assay of two UAS-p35 lines with GS255B driver at 25C *									
8-1	-	*w/Y; 255B/+; +*	M	121	62.33±18.12	68	---------	---------	---------
	L	*w/Y; 255B/+; +*	M	119	62.57±16.22	68	0.39	0	0.478
	L1-10	*w/Y; 255B/+; +*	M	120	66.02±19.38	72	5.91	5.88	7.82E-04
	-	*w/w; 255B/+; +*	F	123	75.95±9.37	78	---------	---------	---------
	L	*w/w; 255B/+; +*	F	124	69.02±12.88	74	-9.13	-5.13	7.69E-07
	L1-10	*w/w; 255B/+; +*	F	124	78.18±9.17	80	2.93	2.56	7.84E-04
10-1	-	*w/Y; 255B/p35; +*	M	111	56.32±25.51	66	---------	---------	---------
	L	*w/Y; 255B/p35; +*	M	4	16±21.6	7	-71.59	-89.39	6.47E-05
	L1-10	*w/Y; 255B/p35; +*	M	117	58.56±17.95	62	3.98	-6.06	0.528
	-	w/w*; 255B/p35; +	F	119	68.47±13.26	72	---------	---------	---------
	L	w/w*; 255B/p35; +	F	30	27.47±16.58	24	-59.89	-66.67	0
	L1-10	w/w*; 255B/p35; +	F	124	64.5±16.45	70	-5.8	-2.78	0.757
11-1	-	*w/Y; 255B/+; p35/+*	M	117	66.15±9.97	68	---------	---------	---------
	L	*w/Y; 255B/+; p35/+*	M	1	14±NA	14	-78.84	-79.41	3.38E-14
	L1-10	*w/Y; 255B/+; p35/+*	M	123	64.98±15.73	70	-1.78	2.94	0.099
	-	w/w*; 255B/+; p35/+	F	123	74.41±5.98	76	---------	---------	---------
	L	w/w*; 255B/+; p35/+	F	0	NA	NA	---------	---------	---------
	L1-10	w/w*; 255B/+; p35/+	F	123	74.37±11.95	78	-0.04	2.63	0.003
*Exp6 Life span assay of two UAS-p35 lines with Elav driver at 25C*									
8-5	-	*yw/Y; +/+; Elav/+*	M	108	61.69±17.95	67	---------	---------	---------
	L1-10	*yw/Y; +/+; Elav/+*	M	115	54.17±15.55	58	-12.18	-13.43	1.80E-08
	-	*yw/w; +/+; Elav/+*	F	120	57.42±14.79	64	---------	---------	---------
	L1-10	*yw/w; +/+; Elav/+*	F	117	56.41±10.82	58	-1.75	-9.38	0.004
10-5	-	*yw/Y; p35/+; Elav/+*	M	121	46.6±7.2	46	---------	---------	---------
	L1-10	*yw/Y; p35/+; Elav/+*	M	115	37.81±9.05	38	-18.86	-17.39	7.06E-14
	-	yw/w*; p35/+; Elav/+	F	123	50.63±15.32	54	---------	---------	---------
	L1-10	yw/w*; p35/+; Elav/+	F	121	49.19±12.16	50	-2.85	-7.41	0.035
11-5	-	*yw/Y; +; Elav/p35*	M	120	54.32±13.04	56	---------	---------	---------
	L1-10	*yw/Y; +; Elav/p35*	M	111	52.31±10.98	52	-3.7	-7.14	0.037
	-	yw/w*; +; Elav/p35	F	123	52.7±13.97	54	---------	---------	---------
	L1-10	yw/w*; +; Elav/p35	F	118	56.63±10.73	58	7.45	7.41	0.091

**Table 4.** Parameters for Gompertz-Makeham model and likelihood ratio test results.

	**Parameters**	**L**	**-**		**chi2**	**df**	**p Value**	**chi2**	**df**	**p Value**
Females										
		one parameter compared at each time								
p35 (X)								Both a and b are constrained		
	a	5.39 x 10^-9^	3.96 x 10^-7^	1.789		1	0.181			
	b	3.89 x 10^-1^	3.00 x 10^-1^	1.516		1	0.218			
	c	2.08 x 10^-2^	1.63 x 10^-3^	59.967		1	**<0.001**	57.983	1	**<0.001**
									
p35 (2)								b is constrained		
	a	7.71 x 10^-6^	2.10 x 10^-4^	5.234		1	**0.022**	50.203	1	**<0.001**
	b	2.41 x 10^-1^	1.92 x 10^-1^	1.700		1	0.192			
	c	2.52 x 10^-2^	1.37 x 10^-3^	50.610		1	**<0.001**	50.154	1	**<0.001**
									
p35 (3)								Both a and b are constrained		
	a	3.31 x 10^-6^	3.00 x 10^-6^	0.003		1	0.958			
	b	2.50 x 10^-1^	2.46 x 10^-1^	0.009		1	0.923			
	c	1.36 x 10^-2^	1.80 x 10^-4^	46.090		1	**<0.001**	66.787		**<0.001**

## Discussion

The tissue and temporal specificity of transgene expression can have significant effects on *Drosophila* life span, and therefore the ability meaningfully to interpret results depends upon careful characterization of the expression patterns produced by the system chosen to drive transgene expression [17,37]. Here the Geneswitch system driver Act-GS-255B was found to yield tissue-general expression of target transgenes in both larvae and adults, including modulation of expression by titrating the concentration of drug in the food.  Some sex-dependent effects on expression were observed with the Geneswitch drivers. For example, Act-GS-255B produced tissue-general expression in both males and females, however females consistently exhibited higher levels of expression than males.  Poirier et al. have recently reported that the Geneswitch driver S_1_-106 (head fat body) is active in adult females but not males [17], and we found a similar result.  Poirier et al. also reported that the Elav-GS (nervous system) driver had a female bias, but in our experiments the Elav-GS driver supported similar levels of UAS-GFP expression in males and females.  It was particularly striking that while the S_1_-106 and S_1_-32 drivers produced abundant target gene expression in adult fat body, they did not support expression in the larval fat body.

For the Elav-GS driver, previous studies have reported pan-neuronal expression in larvae using a UAS-eGFP reporter [19], nervous system-specific expression in adults using a UAS-eGFP reporter [16], and expression in a subset of neurons in brain and ventral nerve cord in adults using a UAS-LacZ reporter [17].  Here, using the UAS-ultraGFP reporter, Elav-GS was found to produce pan-neuronal staining (i.e., expression in all nervous tissue), plus higher-level expression in a subset of neurons, in both larvae and adults, whereas no expression was observed in any tissues other than nervous system in either larvae or adults.  In contrast, Poirier et al. reported that the Elav-GS driver produced staining in the digestive system (gut) when it was tested with the UAS-LacZ reporter, and that this signal in gut was not induced by drug [17].  One possible explanation for this difference in results is that the endogenous *Drosophila*β-galactosidase is expressed in subregions of the gut [38], and this could have resulted in a background signal when staining for transgenic LacZ activity.  Alternatively, the expression pattern produced by the Elav-GS driver might be affected by culture conditions or genetic background differences.

When the Act-GS-255B ubiquitous driver was used to drive expression of the *p53-259H* transgene in adult flies, it produced life span extension in females, consistent with previous results using the Elav-GS driver [9], and therefore demonstrating that the Act-GS-255B driver can produce increased life span when combined with an appropriate target gene.  Of the fourteen candidate genes tested by over-expression, only a subset caused significant and reproducible effects on life span: *wingless* and *Ras activated form* caused negative effects, while *baculovirus p35 *produced both positive and negative effects depending upon sex and developmental stage for over-expression. Care must be taken when interpreting negative effects on life span, since life span might be decreased due to a novel pathology unrelated to the normal mechanisms modulating life span.  However, that said, it is interesting that these particular genes/pathways were identified from among the set of genes tested.

Over-expression of *wingless* using the tissue-general Act-GS-255B driver was lethal to male and female larvae, and when expressed in adult flies *wingless* dramatically decreased both male and female life span.  In *Drosophila*, *wingless* signaling promotes maintenance of the gut stem cells [39,40] and somatic stem cells in the ovary [41]. Interestingly, the *wingless* homolog Wnt and the Wnt signaling pathway have been implicated in modulating aging-related cellular phenotypes in mammals [42]:  Wnt signaling is implicated in tissue homeostasis and the maintenance of adult stem cell populations in younger mammals, while conversely Wnt signaling is implicated in promoting senescence of muscle stem cells in aging mammals [43]  Moreover, the *Klotho* gene appears to function by inhibiting Wnt signaling, and *Klotho* mutation produces an accelerated aging-like phenotype in mice [44], consistent with a pro-aging effect of the Wnt pathway.  *Drosophila* stem cell populations show defects in replicative homeostasis during aging in the gut [45,46] and gonads [47-50], however it is currently unknown to what extent alterations in stem cell function might limit adult *Drosophila* life span.  It will be of interest to determine if *wingless* over-expression reduces adult fly life span by disrupting the function of one or more stem cell populations, and to further explore the role of *wingless* signaling in the maintenance of stem cell populations during *Drosophila* aging.

Over-expression of *Ras activated form* during *Drosophila* larval development was lethal to males and females, and when expressed in adult flies it dramatically decreased both male and female life span.  Ras signaling has been found to shorten life span and promote cellular senescence in yeast and mammals [51-56], whereas in contrast Ras signaling is reported to promote longevity in long-lived *C. elegans Daf-2* insulin-like receptor mutants [57].  It will be of interest in the future to test in what tissue *Ras activated form* acts to decrease adult fly life span and to determine if this might result from an induction of cellular senescence.

Over-expression of the caspase inhibitor *baculovirus p35* in adult flies using the tissue-general Act-GS-255B driver had little to no effect on life span, using three independent *baculovirus p35* transgenes.  In addition, over-expression of the caspase inhibitor *DIAP1* in adults had no consistent effects on life span. While caution must be exercised in interpreting a negative result, it would tend to suggest that adult fly life span is not limited by a canonical caspase-dependent apoptotic pathway. Relevant to this idea, the apoptotic events in aging rat skeletal muscle are reported to be relatively caspase-independent [6].  When *baculovirus p35* was expressed during larval development using the tissue-general Act-GS-255B driver, it caused reduced mean life span in the resultant male and female adult flies, consistent with the requirement for regulated apoptosis in normal fly development.  However, the female adults that resulted from tissue-general *baculovirus p35* over-expression during development exhibited an unusual bi-phasic survival curve that included a subset of adult females with increased life span.  This bi-phasic curve and subset of long-lived females was not observed with nervous-system expression of *baculovirus p35* in larvae using the Elav-GS driver, suggesting that nervous-tissue may not be the critical tissue; however, these experiments were confounded by toxic effect of the Elav-GS driver itself in drug-treated larvae. It will be of interest in the future to determine what might be the mechanism by which *baculovirus p35* over-expression in larvae produces a subset of females with increased life span, and if it might result from the inhibition of apoptosis in some critical tissue during female development.

## Methods

Drosophila strains. All the target transgenes for over-expression (Table 1) were obtained from Bloomington *Drosophila* Stock Center. The ubiquitous Geneswitch driver lines Act-GS-255B and Act-GS-255A contain multiple copies of a P element construct in which expression of the Geneswitch cDNA is under the control of the tissue-general *Actin5C* promoter [16]. The UAS-ultraGFP strain contain multiple copies of a UAS-eGFP construct, and its construction and characterization have been recently described [18].  The Geneswitch system drivers Elav-GS, MHC-GS, S_1_-32 and S_1_-106 were generously provided by T. Osterwalder and R. Davis [19,20].

Drosophila culture. *Drosophila* culture and life span assays were performed as described previously [16]. GeneSwitch virgins were used in the crosses with males of other lines, with the exception of strains in which the target transgene for over-expression was on the X chromosome.  Life span assays consisted of ~25 flies per vial, and a total 5 vials for each cohort.  For survival assays performed at 25^o^C, flies were transferred to new vials ever other day. For survival assays preformed at 29^o^C flies were transferred to new vials every other day during the first 30-40 days, and then every day for the remainder of the life span. RU486 (Mifepristone, Sigma) was dissolved in ethanol (100%) to make a stock solution of 3.2mg/ml. For adult feeding, 50ul RU486 stock solution was added to the surface of each vial to produce a final concentration of ~160ug/ml; 50ul ethanol was added to the control vials. For larval feeding, 0.5ml of 3.2mg/ml RU486 stock solution (or the indicated diluted concentration) was added to the surface of each bottle to produce a final concentration of ~160ug/ml (or indicated diluted concentration); 0.5ml ethanol was added to control bottles.

GeneSwitch driver characterization ***.*** Adult flies were cultured in vials in the presence and absence of drug for two weeks prior to dissection. Adult male and female flies, head in half, body in half, midgut and hindgut, ovary and testes, were photographed. Larvae at 1^st^ instar, 2^nd^ instar and 3^rd^ instar, as well as 3^rd^ instar dissected tissues (brain, midgut and hindgut, salivary gland, imaginal discs, and fat body) were also photographed. The Leica MZ FLIII fluorescence stereomicroscope together with the SPOT software were used for photographs: The GFP pictures were taken under the fluorescent light with exposure time 4 sec and a gain of 2.

Statistical analysis. Mean, standard deviation, median, percent change in mean, percent change in median, and log rank *p* value were calculated using R 2.6.2 [58]. Analysis of mortality rate was performed with the *WinModest* statistical package [59]. In the Gompertz-Makeham model, the increaseof mortality (μ*_x _*) with age (*x*) is expressed as: μ*_x_*= *a*e*^bx^*+c, where the constant *a* is the initial mortalityrate, *b* is the rate of exponential increase in mortality, and *c* is the age-independent mortality.  The age specific mortality rate (μ*_x_*) was calculated using *WinModest* by binning the days over which deaths were counted (since fly deaths were recorded every other day) such that μ*_x_* = (-ln(N_*x *+ __δ_*_x_* / N*_x_* )) / δ*_x_*(or  P*_x_* = N_*x *+ __δ_*_x_* / N*_x_* and μ*_x_* = -1/δ*_x_* ln(P*_x_* )), where N*_x_*is the number of flies alive at day *x* and δ*_x_* is the bin size [2]. Parameters (*a, b, c*) were also calculated based on a likelihood ratio test. The full model (*a*e*^bx^*+c) was plotted, and the Gompertz-only component (*a*e*^bx^*) was used to build the decomposed survival curves, using μ*_x_*: μ*_x_*= *a*e*^bx^*, P*_x_*= e^-μ*x*^. For the decomposed survival curves, any value below 0.5% survival was considered to be the final data point.
